# Effect of folic acid and vitamin B12 plus rosuvastatin in the treatment of coronary heart disease combined with hyperlipidemia

**DOI:** 10.3389/fcvm.2025.1604862

**Published:** 2025-07-30

**Authors:** Hao Wang, Tiezheng Wang

**Affiliations:** Department of Cardiology, Daqing Oilfield General Hospital, Daqing, Heilongjiang, China

**Keywords:** coronary heart disease, hyperlipidemia, folic acid, vitamin B12, rosuvastatin, cardiac function, blood lipid

## Abstract

**Objective:**

To explore the effect of folic acid and vitamin B12 plus rosuvastatin in treating coronary heart disease (CHD) complicated with hyperlipidemia.

**Methods:**

One hundred CHD patients combined with hyperlipidemia admitted to our hospital from January 2022 to December 2023 were separated into study group as well as control group. The control group accepted rosuvastatin calcium tablets. The study group received rosuvastatin plus folic acid and vitamin B12. The cardiac function, blood lipid, plasma Hcy, folate and vitamin B12 levels, inflammation, clinical effect along with occurrence of adverse reactions were compared in 2 groups.

**Results:**

Compared to before therapy, LVEDD and LVESD levels were declined while LVEF level was elevated in 2 groups after therapy, TC, TG and LDL-C levels were declined while HDL-C level was elevated in 2 groups after therapy, plasma Hcy level was declined while folate and vitamin B12 levels were elevated in 2 groups after therapy, and IL-6, TNF-α along with CRP levels were declined in 2 groups after therapy. Compared to control group, the improvements of the above cardiac function indicators, blood lipid indexes, plasma Hcy, folate and vitamin B12 levels, serum inflammatory markers in the study group were more apparent, the total effective rate in the study group presented better. No difference was seen in the occurrence of adverse reactions between 2 groups.

**Conclusion:**

Folic acid and vitamin B12 plus rosuvastatin can effectively improve cardiac function, blood lipid, inflammatory response, and has high safety in treating CHD complicated with hyperlipidemia.

## Introduction

Hyperlipidemia refers to a condition where the levels of cholesterol, triglycerides, phospholipids and non-free fatty acids in the blood are higher than the normal range (1). It is one of the most common types of lipid metabolism disorders in clinical practice, and it is a major risk factor for coronary artery disease, with a hereditary component (2). As a chronic inflammatory disease caused by abnormal lipid metabolism, the global incidence and mortality rate of coronary heart disease (CHD) continue to rise (3). By 2021, the number of CHD in China had reached 11.39 million (4). Studies have shown that hyperlipidemia increases the risk of developing CHD (5). Clinically, the combination of CHD and hyperlipidemia is quite common. The condition is more severe, the course of the disease is longer, and the quality of life of the patients is lower, which will affect the compliance with treatment (6).

Statins are the main drugs in the treatment of CHD complicated with hyperlipidemia (7). Rosuvastatin calcium is a newly developed statin that can reduce low-density lipoprotein cholesterol (LDL-C) and total cholesterol (TC) and prevent the occurrence and development of CHD (8). Rosuvastatin calcium specifically represses 3-hydroxy-3-methyl-glutaryl-coenzyme A (HMG-CoA) reductase, delays or prevents the development of atherosclerosis, and can reduce certain inflammatory factors (9). However, the effect of simple use of this drug is limited, and the long-term curative effect is not ideal (10).

Folic acid and vitamin B12 are essential micronutrients during human growth and development. They are involved in the normal development of nerves, blood vessels, and cells, as well as the maintenance of their functions (11). The absence of these two factors and metabolic abnormalities are risk factors for many diseases. Metabolic abnormalities can lead to the accumulation of precursor substances such as cysteine and methylmalonic acid, which disrupt the stability of blood vessels and nerves and cause impaired cellular function (12).

In our study, we aimed to explore the effect of folic acid and vitamin B12 plus rosuvastatin in treating CHD complicated with hyperlipidemia.

## Materials and methods

### General data

This was a randomized controlled trial. One hundred CHD patients combined with hyperlipidemia admitted to our hospital from January 2022 to December 2023 were chosen and randomly separated into study group (*n* = 50) as well as control group (*n* = 50). Inclusion criteria: (1) Patients met the diagnostic criteria of CHD combined with hyperlipidemia; (2) The clinical data of the patients were complete; (3) Informed consent forms were signed by patients and their families. Exclusion criteria: (1) Patients with abnormal liver function and renal function; (2) Patients with malignant tumors; (3) Patients with autoimmune disease, stroke, cognitive impairment, or other serious medical conditions; (4) Patients with allergic reactions to the drugs used in this study; (5) Patients who had taken hormones or other drugs that could affect the results of this study within the month prior to this study; (6) Patients who withdrew from the study halfway; (7) Patients who did not comply with the treatment. This study was approved by the Ethics committee of Daqing Oilfield General Hospital (Ethical Approval Number: 20220111A). Ethical approval covered all operational procedures and complied with the provisions of the Helsinki Declaration. Informed consent was obtained from all participants prior to their inclusion in the study. Our study has been registered with the Chinese Clinical Trial Registry (ChiCTR), with trial registration number ChiCTR2300072425.

### Sample size

Sample size was calculated using G*Power software (13). Assuming *P*-value for significant of less than 0.05 (two-tailed) and the power of 0.80, the calculated sample size was 100, with 50 cases in each group.

### Randomization and blinding

The patients were randomly assigned to each group in a 1:1 ratio, and this was achieved through computer-generated random numbers. The random allocation table was prepared by a statistician, and this process did not involve any participation of the research team. No stratification factors were used. The research team received a list of blind, sequential packaging identification numbers, and after being included in the study, the patients would be sequentially assigned to the next packaging number on the list. This study was double-blind, and all researchers and patients kept the allocation of groups confidential.

### Treatment methods

The control group took rosuvastatin calcium tablets (Nanjing Zhengda Tianqing Pharmaceutical Co., LTD.) orally, 10 mg each time, once a day.

The study group took rosuvastatin plus folic acid and vitamin B12. The usage of rosuvastatin calcium tablets was the same as the control group. Besides, patients took folic acid (Hangzhou Aoyipollen Pharmaceutical Co., LTD.) orally, 0.4 mg/time, once/day. At the same time, patients took vitamin B12 (Xi'an Disai Biological Pharmaceutical Co., LTD.) orally, 25 μg/time, 3 times/day. The dosages of folic acid and vitamin B12 were adopted according to the standards stipulated in clinical guidelines.

Both groups accepted treatment for 3 months.

### Observation indicators

(1) Cardiac function indicators: Left ventricular ejection fraction (LVEF), left ventricular end diastolic diameter (LVEDD) along with left ventricular end systolic diameter (LVESD) were tested by echocardiography.
(2) Blood lipid index: Serum levels of TC, triglyceride (TG), high density lipoprotein cholesterol (HDL-C) and LDL-C were tested by automatic biochemical analyzer.
(3) Plasma homocysteine (Hcy), folate and vitamin B12 levels: Automatic biochemical analyzer was implemented to detect the Hcy level, and an automatic chemiluminescence instrument was used to detect the folic acid and vitamin B12 levels.
(4) Serum inflammatory markers: Serum levels of interleukin-6 (IL-6), tumor necrosis factor-α (TNF-α) along with C-reactive protein (CRP) were tested by enzyme-linked immunosorbent assay.
(5) Clinical effect: After the treatment, the frequency of angina pectoris attacks decreased by more than 75%, the NYHA cardiac function classification improved by 2 levels or more, the TC decreased by ≥20% and the TG decreased by ≥40%, which was markedly improved; After treatment, the frequency of angina pectoris attacks decreased by 50%-75%, the NYHA cardiac function classification improved by 1 level, the TC decreased by 10%-20% and the TG decreased by 20%-40%, which was effective; After the treatment, the frequency of angina pectoris attacks decreased by less than 50%, the NYHA cardiac function classification did not improve, the TC showed a decrease of less than 10% and the TG showed a decrease of less than 20%, which was ineffective. Total effective rate = (number of markedly improved cases + number of effective cases)/Total number of cases ×100%.
(6) The occurrence of adverse reactions (nausea and vomiting, gastrointestinal discomfort, dizziness, and palpitation) was recorded.

### Statistical analysis

SPSS 24.0 statistical software was adopted for data analysis. Measurement data were expressed as (x ± s), and *t*-test was adopted for comparison. Count data were expressed as (*n*, %), and χ^2^ test was adopted for comparison. *P* < 0.05 meant statistical significance.

## Results

### General data of patients in 2 groups

No difference was discovered in general data between 2 groups (*P* > 0.05, Table 1).

**Table 1:** General data of patients in 2 groups.

Groups	Cases	Gender		Age (years)	Course of disease (years)
		Male	Female		
Control group	50	28 (56.00)	22 (44.00)	57.12 ± 7.42	7.12 ± 3.55
Study group	50	27 (54.00)	23 (46.00)	57.18 ± 7.46	7.15 ± 3.58
χ^2^/*t*		0.040		0.040	0.042
*P*		0.840		0.967	0.966

### Cardiac function indicators in 2 groups

Compared to before therapy, LVEDD and LVESD levels were declined while LVEF level was elevated in 2 groups after therapy (*P* < 0.001). Compared to control group, the improvements of the above cardiac function indicators in the study group were more significant (*P* < 0.001, Table 2).

**Table 2:** Cardiac function indicators in 2 groups.

Indicator	Control group (*n* = 50)	Study group (*n* = 50)	*t*	*P*
LVEDD (mm)				
Pre-treatment	54.97 ± 5.62	55.06 ± 5.51	0.080	0.935
Post-treatment	50.23 ± 5.02	45.15 ± 4.53	5.312	<0.001
*t*	4.447	9.823		
*P*	<0.001	<0.001		
LVESD (mm)				
Pre-treatment	44.85 ± 4.52	44.97 ± 4.49	0.133	0.894
Post-treatment	40.36 ± 4.32	34.58 ± 3.42	7.417	<0.001
*t*	5.077	13.016		
*P*	<0.001	<0.001		
LVEF (%)				
Pre-treatment	37.78 ± 3.82	37.71 ± 3.75	0.092	0.926
Post-treatment	41.26 ± 4.16	48.89 ± 4.92	8.373	<0.001
*t*	4.356	12.779		
*P*	<0.001	<0.001		

### Blood lipid indexes in 2 groups

Compared to before therapy, TC, TG and LDL-C levels were declined while HDL-C level was elevated in 2 groups after therapy (*P* < 0.001). Compared to control group, the improvements of the above blood lipid indexes in the study group were more significant (*P* < 0.001, Table 3).

**Table 3:** Blood lipid indexes in 2 groups.

Indicator	Control group (*n* = 50)	Study group (*n* = 50)	*t*	*P*
TC (mmol/L)				
Pre-treatment	5.85 ± 0.58	5.82 ± 0.57	0.260	0.794
Post-treatment	4.23 ± 0.43	3.12 ± 0.32	14.643	<0.001
*t*	15.865	29.206		
*P*	<0.001	<0.001		
TG (mmol/L)				
Pre-treatment	1.96 ± 0.21	1.95 ± 0.19		
Post-treatment	1.23 ± 0.12	0.92 ± 0.09		<0.001
*t*				
*P*	<0.001	<0.001		
HDL-C (mmol/L)				
Pre-treatment	1.23 ± 0.13	1.20 ± 0.12	1.199	0.233
Post-treatment	1.45 ± 0.15	1.76 ± 0.18	9.355	<0.001
*t*	7.837	18.304		
*P*	<0.001	<0.001		
LDL-C (mmol/L)				
Pre-treatment	3.64 ± 0.37	3.61 ± 0.36	0.410	0.682
Post-treatment	3.02 ± 0.32	2.73 ± 0.27	4.897	<0.001
*t*	8.962	13.827		
*P*	<0.001	<0.001		

### Plasma Hcy, folate and vitamin B12 levels in 2 groups

Compared to before therapy, plasma Hcy level was declined while folate along with vitamin B12 levels were elevated in 2 groups after therapy (*P* < 0.001 and *P* = 0.003). Compared to control group, the improvements of plasma Hcy, folate along with vitamin B12 levels in the study group were more significant (*P* < 0.001, Table 4).

**Table 4:** Plasma Hcy, folate and vitamin B12 levels in 2 groups.

Indicator	Control group (*n* = 50)	Study group (*n* = 50)	*t*	*P*
Hcy (μmol/L)				
Pre-treatment	15.21 ± 1.52	15.24 ± 1.52	0.098	0.921
Post-treatment	12.36 ± 1.35	8.01 ± 0.82	19.473	<0.001
*t*	9.912	29.601		
*P*	<0.001	<0.001		
Folate (ng/ml)				
Pre-treatment	419.65 ± 42.15	420.05 ± 42.69	0.047	0.962
Post-treatment	445.62 ± 44.52	536.27 ± 53.28	9.232	<0.001
*t*	2.995	12.037		
*P*	0.003	<0.001		
Vitamin B12 (pg/ml)				
Pre-treatment	200.65 ± 20.21	202.41 ± 20.15	0.436	0.663
Post-treatment	256.38 ± 25.78	326.21 ± 32.68	22.801	<0.001
*t*	12.029	11.862		
*P*	<0.001	<0.001		

### Serum inflammatory markers in 2 groups

Compared to before therapy, IL-6, TNF-α along with CRP levels were declined in 2 groups after therapy (*P* < 0.001). Compared to control group, the reduction of the above serum inflammatory markers in the study group were more significant (*P* < 0.001, Table 5).

**Table 5:** Serum inflammatory markers in 2 groups.

Indicator	Control group (*n* = 50)	Study group (*n* = 50)	*t*	*P*
IL-6 (ng/L)				
Pre-treatment	24.86 ± 2.52	24.89 ± 2.49	0.059	0.952
Post-treatment	14.21 ± 1.43	8.08 ± 0.81	26.374	<0.001
*t*	25.990	45.395		
*P*	<0.001	<0.001		
CRP (mg/L)				
Pre-treatment	6.75 ± 0.69	6.78 ± 0.68	0.219	0.827
Post-treatment	4.35 ± 0.45	3.01 ± 0.32	17.159	<0.001
*t*	20.601	35.471		
*P*	<0.001	<0.001		
TNF-α (mg/L)				
Pre-treatment	1.78 ± 0.18	1.81 ± 0.19	0.810	0.419
Post-treatment	1.05 ± 0.11	0.68 ± 0.07	20.066	<0.001
*t*	24.469	39.461		
*P*	<0.001	<0.001		

### Clinical effect in 2 groups

Relative to control group, the total effective rate in the study group was better (*P* = 0.007, Table 6).

**Table 6:** Clinical effect in 2 groups.

Groups	Cases	Markedly improved	Effective	Ineffective	Total effective rate
Control group	50	15 (30.00)	24 (48.00)	11 (22.00)	39 (78.00)
Study group	50	20 (40.00)	28 (56.00)	2 (4.00)	48 (96.00)
χ^2^					7.162
*P*					0.007

### Occurrence of adverse reactions in 2 groups

No difference was seen in the occurrence of adverse reactions between 2 groups (*P* = 0.101, Table 7).

**Table 7:** Occurrence of adverse reactions in 2 groups.

Groups	Cases	Nausea and vomiting	Gastrointestinal discomfort	Dizziness	Palpitation	Total incidence rate
Control group	50	2 (4.00)	3 (6.00)	2 (4.00)	2 (4.00)	11 (22.00)
Study group	50	2 (4.00)	1 (2.00)	1 (2.00)	1 (2.00)	5 (10.00)
χ^2^						2.679
*P*						0.101

## Discussion

Recently, with the continuous growth of the population in our country, the incidence of CHD has also been increasing year by year (14). Dyslipidemia is the main risk factor of CHD (15). Chronic dyslipidemia can damage vascular endothelial cells through a variety of mechanisms, resulting in cardiovascular lipid infiltration, which leads to atherosclerosis (16). Statins can effectively promote the clearance of cholesterol in cells, and are commonly used in clinical routine drugs to treat dyslipidemia (17). Rosuvastatin is a selective reductase inhibitor that acts mainly on the liver of patients and enables the presence of polar methylsulfonamidos in its molecules (18). However, the effect of this single drug is not ideal, and relevant experts believe that the combined use of drugs can effectively enhance the overall treatment outcome (19).

As a sulfur-containing amino acid and an intermediate product of methionine, Hcy is mainly derived from dietary methionine (20). Numerous studies have validated that the elevated serum Hcy level is an independent risk factor for various cardiovascular diseases (21). High serum Hcy level causes direct or indirect damage to vascular endothelial cells, resulting in increased secretion of endothelin by endothelial cells, thus affecting the balance of nitric oxide and endothelin, leading to atherosclerosis (22). Besides, Hcy can also cause endothelial cell damage through oxidative stress response, lead to proliferation of vascular smooth muscle cells, induce platelet aggregation and adhesion, and promote thrombosis (23).

Folic acid and vitamin B12 are both important substances involved in methionine cycle in human Hcy metabolism (24). The traditional view holds that a deficiency in folic acid and vitamin B12 can lead to a significant accumulation of Hcy in the body, which in turn can cause cardiovascular diseases; while appropriate supplementation of folic acid and vitamin B12 can exert cardiovascular protective effects by reducing Hcy levels (25). However, recent studies have found that folic acid and vitamin B12 may be important risk factors for CHD that are independent of Hcy, However, the specific mechanism of their effects is not yet fully understood. This might be related to the dysfunction of vascular endothelial cells caused by folate deficiency. For instance, folate deficiency can increase the inflammatory response of cells, while the production of nitric oxide will increase the risk of cardiovascular diseases (26).

In our study, the results suggested that relative to control group, the improvements of LVEDD, LVESD and LVEF levels in the study group were more significant, suggesting that folic acid and vitamin B12 plus rosuvastatin could improve the cardiac function in treating CHD complicated with hyperlipidemia. The reason is that vitamin B12 and folic acid both belong to the B group of vitamins. They can participate in the synthesis process of methionine and thymine, and can transfer the methyl group from methyl tetrahydrofolate to methyl donors such as Hcy to form methyl Hcy, which reduces the level of Hcy in the blood, thereby avoiding the damage to endothelial function caused by high Hcy, delaying or even reversing the process of atherosclerosis, and improving cardiac function (27). Consistently, Dkhillon et al. demonstrated that the intake of folic acid and vitamin B could decrease the heart rate and increase LVEF in patients diagnosed with chronic heart failure (27).

Our study also revealed that compared to control group, the improvements of TC, TG, HDL-C along with LDL-C levels in the study group were more significant, implying that folic acid and vitamin B12 plus rosuvastatin could improve the blood lipid in treating CHD complicated with hyperlipidemia. Besides, our study indicated that compared to control group, the improvements of plasma Hcy, folate along with vitamin B12 levels in the study group were more significant. The reason is that the content of folic acid and vitamin B12 is negatively correlated with the content of Hcy. When the content of Hcy decreases, the content of folic acid increases. This further enhances the effect of modifying liposomes and inhibiting oxidative stress, thereby improving the lipid abnormality conditions of patients (28). Likewise, Fogacci et al. discovered that folic acid supplementation may improve plasma lipid levels as well as reduce the risk of cerebrovascular disease (29). Boachie et al. suggested that low vitamin B12 increased fatty acid synthesis and levels of individual fatty acids, and decreased fatty acid oxidation and mitochondrial respiration, thus resulting in dysregulation of lipid metabolism (30).

Moreover, our study indicated that compared to control group, the reduction of IL-6, TNF-α as well as CRP levels in the study group were more significant, reflecting that folic acid and vitamin B12 plus rosuvastatin could improve the inflammatory response in treating CHD complicated with hyperlipidemia. The reason is that after folic acid and vitamin B12 enter the body, they inhibit the body's oxidative stress response, promote the restoration of balance and stability of the body's own system, and thereby reduce the release of inflammatory factors such as IL-6 and TNF-α, and alleviate inflammatory damage (31). Similarly, Chen et al. pointed that folic acid and vitamin B12 supplementation could reduce the inflammatory cytokine levels in patients with Alzheimer's disease (11). In addition, our study indicated that relative to the control group, the total effective rate in the study group presented better, which was also supported by previous reports (32, 33). At the same time, no difference was seen in the occurrence of adverse reactions between 2 groups, indicating the safety of the combination use of folic acid, vitamin B12 and rosuvastatin in treating CHD complicated with hyperlipidemia.

There are some limitations of our study. First, the sample size is relatively small (*n* = 100 total), which may limit the generalizability and statistical power for detecting less common adverse events or subtle differences. Second, the study duration of 3 months is relatively short for assessing long-term clinical outcomes, such as cardiovascular events or mortality. Furthermore, there are some potential interfering factors (such as factors related to diet or lifestyle) that were not controlled in our study. Therefore, more large-scale and long-term studies should be conducted in the future to further verify our findings.

In conclusion, our study demonstrates that folic acid and vitamin B12 plus rosuvastatin may effectively improve cardiac function, blood lipid, inflammatory response, and has high safety in treating CHD complicated with hyperlipidemia.

## Data Availability

The datasets presented in this study can be found in online repositories. The names of the repository/repositories and accession number(s) can be found in the article/Supplementary Material.
